# Prognostic value and microenvironmental crosstalk of exosome-related signatures in human epidermal growth factor receptor 2 positive breast cancer

**DOI:** 10.1515/biol-2022-0899

**Published:** 2024-07-24

**Authors:** Ji Zhao, Feng Shen, Yue-Mei Hu, Kai Yin, Ying Chen, Yan-Jie Chen, Qun-Chao Hu, Li Liang

**Affiliations:** Department of Breast Surgery, Tong Ren Hospital, Shanghai Jiao Tong University School of Medicine, Shanghai, 200336, People’s Republic of China; Department of Medical Oncology, Zhongshan Hospital (Xiamen), Fudan University, Xiamen, 361015, People’s Republic of China; Department of Medical Oncology, Zhongshan Hospital Fudan University, No. 180, Fenglin Road, Xuhui District, Shanghai, 200032, People’s Republic of China; Department of Pathology, Tong Ren Hospital, Shanghai Jiao Tong University School of Medicine, Shanghai, 200336, People’s Republic of China; Department of Radiation Oncology, Tong Ren Hospital, Shanghai Jiao Tong University School of Medicine, 1111 Xianxia Road, Changning District, Shanghai, 200336, People’s Republic of China; Department of Gastroenterology, Zhongshan Hospital (Xiamen), Fudan University, No. 668, Jinhu Road, Huli District, Xiamen, 361015, People’s Republic of China; Department of Gastroenterology, Zhongshan Hospital of Fudan University, 180 Fenglin Road, Xuhui District, Shanghai 200032, People’s Republic of China

**Keywords:** breast cancer, exosomes, human epithelial growth factor receptor 2, microenvironment, prognosis, biomarkers

## Abstract

This study aimed to determine the prognostic value and microenvironmental crosstalk of exosome-related signatures in human epidermal growth factor receptor 2 positive breast cancer (HER2^+^ BC). Transcriptome sequencing and clinicopathological data were downloaded from the Cancer Genome Atlas. The 10X single cell sequencing dataset was downloaded from the National Center for Biotechnology Information Gene Expression Omnibus. Exosomes-Related Genes were extracted from the ExoCarta and Gene Set Enrichment Analysis databases. FGF9, SF3B4, and EPCAM were found and deemed the most accurate predictive signatures. Patients with HER2^+^ BC were subtyped into three groupings by exosome prognostic gene (EPGs). The expression of SF3B4 was positively linked with the infiltration of macrophages, neutrophils, and CD4^+^ T cells. The expression characteristics of EPGs were associated with the biological process of “response to xenobiotic stimuli.” Interactions were relatively high between malignant epithelial cells and fibroblasts, endothelial cells, monocytes, and macrophages. Malignant epithelial cells interact more with fibroblasts and endothelial cells. The migration inhibitory factor pathway was the primary outgoing signaling pattern, while the C-C motif chemokine ligand pathway was the primary incoming signaling pattern for communication between malignant epithelial cells and macrophages. This study described the role of exosome signatures in the prognosis and microenvironment of HER2^+^ BC and provided a basis for future research.

## 1 Introduction

Breast cancer is the most prevalent female malignant tumor worldwide. It is predicted that there will be 300,590 new cases of breast cancer and 43,700 deaths in the United States alone in 2023 [1]. The number of new breast cancer cases in China in 2022 was estimated to be 420,000, with breast cancer ranking first and fourth in morbidity and mortality, respectively, among the Chinese population [2]. The overexpressed human epidermal growth factor receptor 2 positive breast cancer (HER2^+^ BC) comprises 15–20% of breast cancer cases and displays sensitivity to anti-HER2-targeted medicines, such as transtuzumab (binding specifically to the HER2 receptor and preventing its activation), pertuzumab (blocking the dimerization of HER2 with other growth factor receptors), lapatinib (inhibiting directly the internal kinase activity of HER2 and epidermal growth factor receptor), and T-DM1 (allowing tertuzumab to deliver chemotherapy directly to HER2-overexpressing tumor cells) [3]. Despite the efficacy of these drugs, primary and secondary resistance frequently develop.

Since HER2^+^ BC is characterized by dense immunological cell infiltration and reduced immune exhaustion [4], immune checkpoint inhibitors combined with standard anti-HER2 drugs have demonstrated success in the treatment of HER2^+^ BC [5]. Exosomes are extracellular vesicles that are 30–150 nm in diameter and originate from the multivesicular endosome pathway. Exosomes are composed of a lipid bilayer and contain DNA, RNA, proteins, and lipids from their parent cells. Exosomes are involved in the internal signal interaction, tumor metastasis, extracellular matrix reconstruction, tumor microenvironment control (cell communication and signaling, immunoregulation, extracellular matrix remodeling, angiogenesis, promoting tumor drug resistance), and drug resistance [6]. Exosome-related genes (ERG) have shown important value in the diagnosis and prognosis evaluation of breast cancer. They affect the tumor microenvironment and cancer progression by transmitting information between cells. HER2^+^ BC is a specific subtype characterized by overexpression of HER2, which is associated with specific clinicopathological features and treatment response. Understanding the role of ERGs in HER2^+^ BC has potential implications for developing new therapeutic strategies and improving patient outcomes. Ciravolo et al. found that HER2-overexpressing breast cancer cell lines exhibited resistance to trastuzumab by releasing HER2-expressed exosomes into the extracellular environment [7]. Barok et al. discovered that T-DM1 may bind to HER2^+^ BC-derived exosomes and be transferred to additional cancer cells by the exosomes, thereby inhibiting the survival of target cells [8]. Limoni et al. created a drug delivery method by engineering exosomes and delivering siRNA to HER2^+^ BC cells [9].

Thus, we hypothesize that exosomes may serve as a predictive biomarker for HER2^+^ BC and play a crucial role in the interaction between HER2^+^ BC and the microenvironment. However, there are insufficient reports on this topic.

In this study, we identified the differentially expressed genes (DEGs) in HER2^+^ BC and ERGs, determined the exosome prognostic genes (EPGs) via Kaplan–Meier (KM) analysis, LASSO Cox regression, and random forest model, performed the clustering analysis, mutation feature analysis, enrichment analysis, and determined the relationship between EPGs and the immune microenvironment using a single cell RNA-sequencing dataset containing five clinical samples. Our findings not only validated the markers that predict the clinical prognosis of HER2^+^ BC from the perspective of exosomes, but also provide a research foundation for establishing the link between exosome signatures and the immunological microenvironment in HER2^+^ BC.

## 2 Methods

### 2.1 Raw data acquisition

We acquired the breast cancer transcriptome sequencing data and clinicopathological information from The Cancer Genome Atlas (TCGA; https://portal.gdc.cancer.gov/) database [10] through the R program TCGAbiolink [11]. All downloaded sample data met the following criteria: (a) cases with complete mRNA expression data and clinical data: ensure that each case selected for the study has sufficient information for subsequent statistical analysis and biological interpretation; (b) by pathological diagnosis of breast cancer: ensure that the research focuses on a specific disease group, and to enhance the pertinence and accuracy of the results; (c) cases accepted standardized treatment of breast cancer, including surgery, chemotherapy, and radiotherapy: select standard treatment cases, helps to reduce the deviation caused by different treatment results; (d) cases of survival time for more than 30 days: eliminate survival cases in a very short time, these cases may be unrepresentative due to special circumstances; and (e) HER2^+^ BC samples can be extracted from all datasets: focus on HER2-positive breast cancer, a patient population with specific biological characteristics and treatment response. The transcriptome data were reannotated using an annotation file from UCSC Xena (https://xenabrowser.net/datapages) for future analysis [12]. Finally, 74 cases of HER2^+^ BC and 99 cases of precancerous normal samples were included. Somatic mutation data on HER2^+^ BC (*n* = 74) were collected from “Masked Somatic Mutation” on the TCGA GDC website (https://portal.gdc.cancer.gov/), preprocessed with VarScan software, and visualized using maftools R package [13]. The single cell sequencing (scRNA seq) dataset for combined analysis was downloaded from the National Center for Biotechnology Information Gene Expression Omnibus (https://www.ncbi.nlm.nih.gov/geo/) [14]. GSE176078 was derived from human DNA and detected on the GPL18573 IIIumina NextSeq 500. The data derived from the primary lesion of 26 patients with breast cancer, including five patients with HER2^+^ BC were assessed in this study (Table 1). ERGs were referenced from the ExoCarta database [15] and the gene set enrichment analysis (GSEA, https://www.gsea-msigdb.org/) [16]. We enlisted a total of 121 ERGs after combination and de-duplication (Table S1).

**Table 1:** Published transcriptome database information

	TCGA-BRCA (HER2^+^)	GSE176078
Platform	Illumina	GPL18573
Species	*Homo sapiens*	*Homo sapiens*
Tissue	Breast cancer	Breast cancer
Samples in cancer group	74	5
Samples in normal group	99	0
Reference	—	PMID: 34493872

### 2.2 Gene set variation analysis (GSVA)

GSVA was performed on HER2^+^ BC from TCGA-BRCA using the R GSVA package (version 1.42.0) [17].

### 2.3 Identification of exosome-related differentially expressed genes (ER-DEGs) in HER2^+^ BC

The R limma package was used to screen DEGs between HER2^+^ BC samples and precancerous TCGA-BRCA samples. The absolute value of Log_2_ [Fold change] (Log_2_ FC) > 1 and *p* < 0.05 were set as the threshold of DEGs. The genes with Log_2_ FC > 1 and *p* < 0.05 were DEGs with up-regulated expression, while the genes with Log_2_ FC < −1 and *p* < 0.05 were DEGs with down-regulated expression. The DEGs were displayed using volcano plots and heat maps.

### 2.4 Identification of EPGs in HER2^+^ BC

Univariate and multivariate Cox regression analyses were first conducted to determine ER-DEGs, followed by the Least Absolute Shrinkage and Selection Operator (LASSO) regression and random forest model to filter the findings of multivariate Cox regression. LASSO Cox model is a statistical technique that combines the LASSO method with the Cox proportional hazard model, which is widely used in survival analysis, especially when dealing with high-dimensional data (such as gene expression data), aiming to identify variables (such as genes or biomarkers) that are significantly correlated with patient survival time. The candidates identified by the two models were plotted and intersected using a Venn Diagram. The dimensionality reduction screening was performed by 1,000 iterations with ten folds to cross-validate the LASSO Cox model [18]. The random forest model was simulated 1,000 times, and the significance of the final results was determined and then ranked. ERGs with an importance greater than 0.2 were chosen as the results. The intersection of genes screened by the two preceding models was considered as the EPGs in HER2^+^ BC. We created a Spearman’s analysis, a heat map, a scatter plot, and a correlation curve drawing using the R package cowplot. Correlation analysis of key genes was performed using the R package GOSemSim [19]. The chromosome localization map was drawn and displayed using the R package RCircos [20].

### 2.5 Mutation analysis of EPGs in HER2^+^ BC

The breast cancer MuTect2 file for Whole Exome Sequencing Mutation Annotation Format was obtained from the TCGA GDC website (https://portal.gdc.cancer.gov/). All non-synonymous mutations were chosen for downstream analysis. EPG mutations in HER2^+^ BC were exhibited through R package maftools [21].

### 2.6 Construction of prognostic predictive model by EPGs in HER2^+^ BC

The nomogram graphic, calibration analysis, and calibration curve drawing were completed using the R package rms [22]. Next, a calibration analysis was run and a calibration curve built to evaluate the accuracy and resolution of the nomogram. We created the time-dependent receiver operative characteristic (ROC) curves to assess the accuracy of the prognostic model for the 1-, 3-, and 5-year survival outcomes for HER2^+^ BC using the R package survivalROC [23]. Decision curve analysis (DCA) maps were created to evaluate the accuracy and resolution of Cox regression models using the R package ggDA [24].

### 2.7 Determination of exosome-related subgroups of HER2^+^ BC

Populations with varied exosome functional phenotypic characteristics based on previous EPGs in HER2^+^ BC were found using the consensus clustering method of CONSENSUSClusterPlus in R [25]. Principal coordinates analysis (PCoA) was performed to confirm the consistency clustering results [26]. The box charts were created using the R package ggpubr to group the sample clustering labels. The significant differences between the groups were discovered using the Wilcoxon rank-sum test, with *p*-values less than 0.05 considered statistically significant.

### 2.8 Evaluation of biological characteristics and tumor microenvironment of functional phenotypes of different exosomes in HER2^+^ BC

The Wilcoxon rank-sum test was performed to compare the difference in tumor mutational burden and microsatellite instability (MSI) between different exosome functional phenotypes in HER2^+^ BC, and the results are displayed in a box graph. The TIMER algorithm was used to assess the entrance status of immune cells, and the CIBERSORT algorithm was utilized to assess the difference in immune cell infiltration between HER2^+^ BC samples and normal samples [27]. The TIMER algorithm evaluates the status and activity of immune cells by estimating the proportion of each immune cell in the tumor microenvironment through a deconvolution method using specific immune cell marker genes. The CIBERSORT algorithm provides a powerful tool to explore immune infiltration and immune microenvironment in HER2^+^ BC and other cancers by analyzing a set of gene expression signatures linked to specific cell types.

Single sample gene set enrichment analysis (ssGSEA) was used to perform GSEA and estimate the relative abundance of each immune cell infiltration [28]. The distribution of immune cell infiltration in different risk groups and disease subtypes of HER2^+^ BC was displayed using the R package ggplot2. Correlation heatmaps were created to show the relationship between EPGs, key genes connected to exosomes, and immune cells in different risk categories using the R package heatmap.

### 2.9 Quality control, cluster analysis, cell annotation of single cell data, scoring of ERG sets, and analysis of intercellular communication networks

The Seurat objects for this analysis were created, and quality check was performed by importing the count matrix of five samples from the single cell dataset GSE176078 using the R package Seurat (version 4.0). The standards are listed as follows: (1) The number of genes expressed in cells could not be less than 250. (2) 20% or more unique molecular identifiers (UMIs) were located on mitochondrial or ribosome gene cells. (3) The outlier was filtered by cutting the gene expression to 2 × (mean ± standard deviation). If they met one of the criteria, they were eliminated. To check double cells, the R program DoubletFinder was used with the default parameter settings [29]. The batch effect between samples was corrected using the R package Harmony [30]. After quality standardization and conversion to Seurat objects, the first 2,000 genes with significant variations in expression levels were extracted using the FindVariableFeature function. Dimensionality reduction analysis was implemented using principal component analysis (PCA) based on the expression levels of the 2,000 previously selected genes. Cell clustering analysis was conducted using the FindClusters (with the resolution parameter set to 0.8) and FindAllMarkers functions. To identify the marker genes for each cluster, a cutoff threshold of *p* value <0.05 and a multiple of differences >0.5 were applied. Following the preceding stages, we obtained 48,785 cells. Standard cell types were annotated using the HumanPrimaryCellAtlasData dataset from the R package SingleR (version 1.8.1) [31]. Nine cell types were identified such as T cells, B cells, endothelial cells, epithelial cells, fibroblasts, monocytes, NK cells, dendritic cells (DC), and macrophages by referencing the expression patterns of the marker gene in the GSE176078 and CellMarker datasets [32,33]. Malignant epithelial cells were identified by changes in copy number variation using the CopyKat R program [34]. “Malignant epithelial cells” appraisal aims to identify cancer cells in single cell data and that for us to study the tumor microenvironment, the heterogeneity of cancer cells and immune cells around the interaction is of great significance. Exosomes based on the GSE176078 dataset for scoring were obtained using the Area Under the Curve Cell (AUCell) R package (version 1.12.0) [35]. The set of exosome genes was organized as input data for determining the value of the area under the curve (AUC). A gene expression ranking was established for each cell based on the AUC value. The AUC was used to estimate the proportion of highly expressed genes in each cell. The AUCell_ explore threshold function was used to generate a threshold for evaluating the activity of the present gene set. Then, based on the AUC score of each cell, Uniform Manifold Approximation and Projection (UMAP) encapsulation of the cell clusters was stained to reveal which cell clusters were active in the ERGs. Intercellular communication networks from single-cell RNA sequencing data were inferred statistically and examined using the R package CellChat [36]. Intended cell contact refers to the expected or potential cell-to-cell contact in the analysis of single-cell data, which is usually mediated by specific receptor–ligand pairings. Receptor and the ligand matching is one of the basic mechanisms of intercellular communication, through the combination of cell surface receptors and ligands, transmit signals to promote interaction between cells, the cell behavior and the regulation of physiological status is of great significance. The cell contact of HER2^+^ BC single cell subsets was depicted using a circle diagram. All receptor–ligand pairings during intercellular signal transduction were counted using bubble plots.

### 2.10 Statistical analysis

All data processing and analysis in this study were conducted using R (Version 4.2.0). Continuous variables are presented as the mean ± standard deviation. The comparison between the two groups was conducted using the Wilcoxon rank-sum test. Three or more groups were compared using the Kruskal–Wallis test. The Kruskal–Wallis test is a generalization of the Wilcoxon rank-sum test on groups of three or more independent samples. It was used to test multiple sample group median whether there was a statistically significant difference, which did not have to require the same data of normal distribution. This method is a non-parametric approach to assess whether multiple groups have the same distribution on a continuous variable. The chi-squared test or the Fisher’s exact test was utilized to compare and analyze the statistical significance between two sets of categorical variables. GSVA analysis was performed on the TCGA-BRCA samples obtained after integration through the EPGs. Exosome function-related scores of HER2^+^ BC in TCGA-BRCA were calculated. To investigate the association between exosome function and clinical phenotype, Wilcoxon analysis was used. KM analysis was used to evaluate the prognosis. Spearman’s correlation analysis was used to determine correlation coefficients between different genes. *p* Value of less than 0.05 was considered as the criterion for statistically significant differences.

## 3 Results

### 3.1 Identifying EPGs in patients with HER2^+^ BC

**Figure 1:**
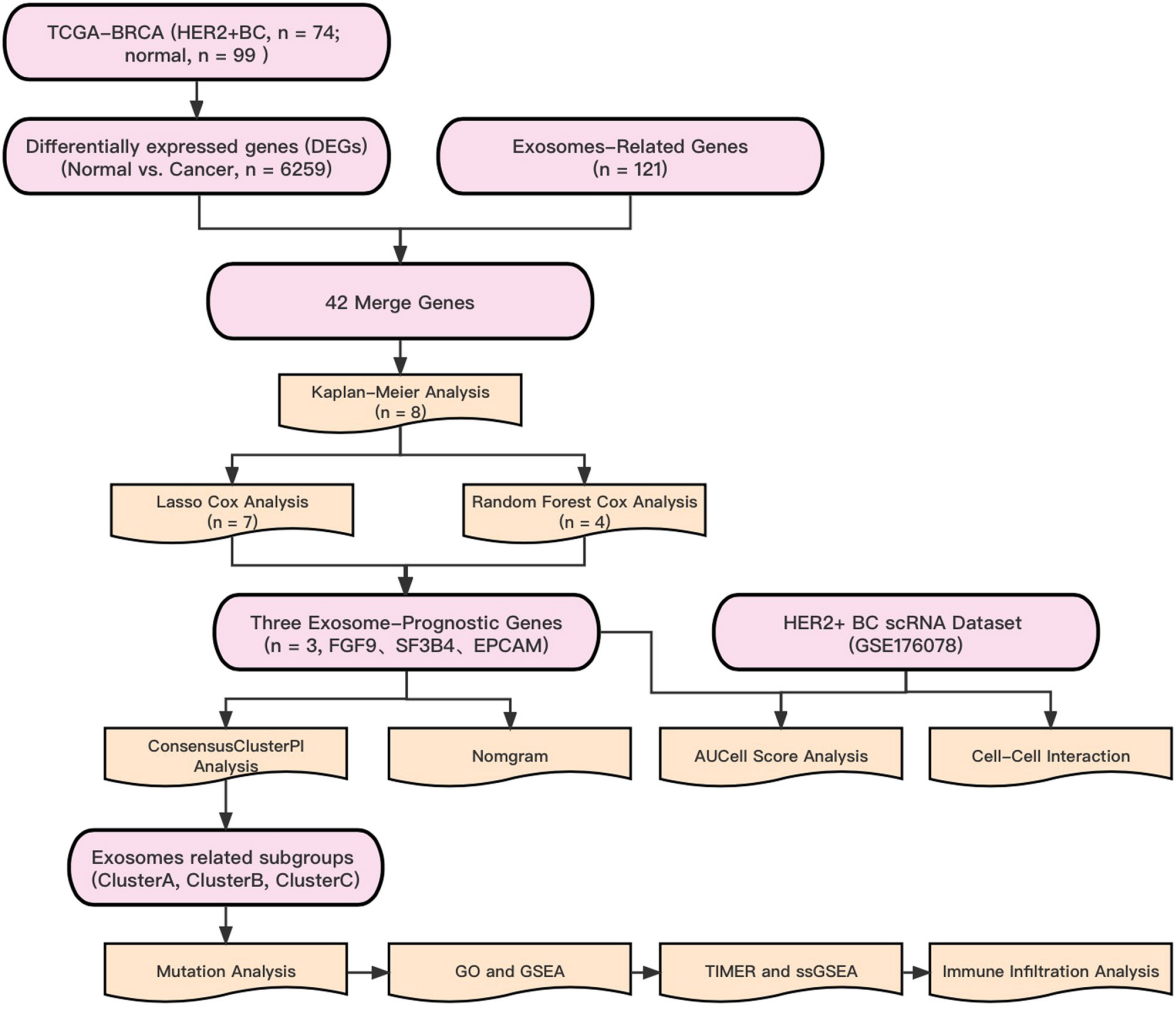
Work flowchart of this study.

**Figure 2:**
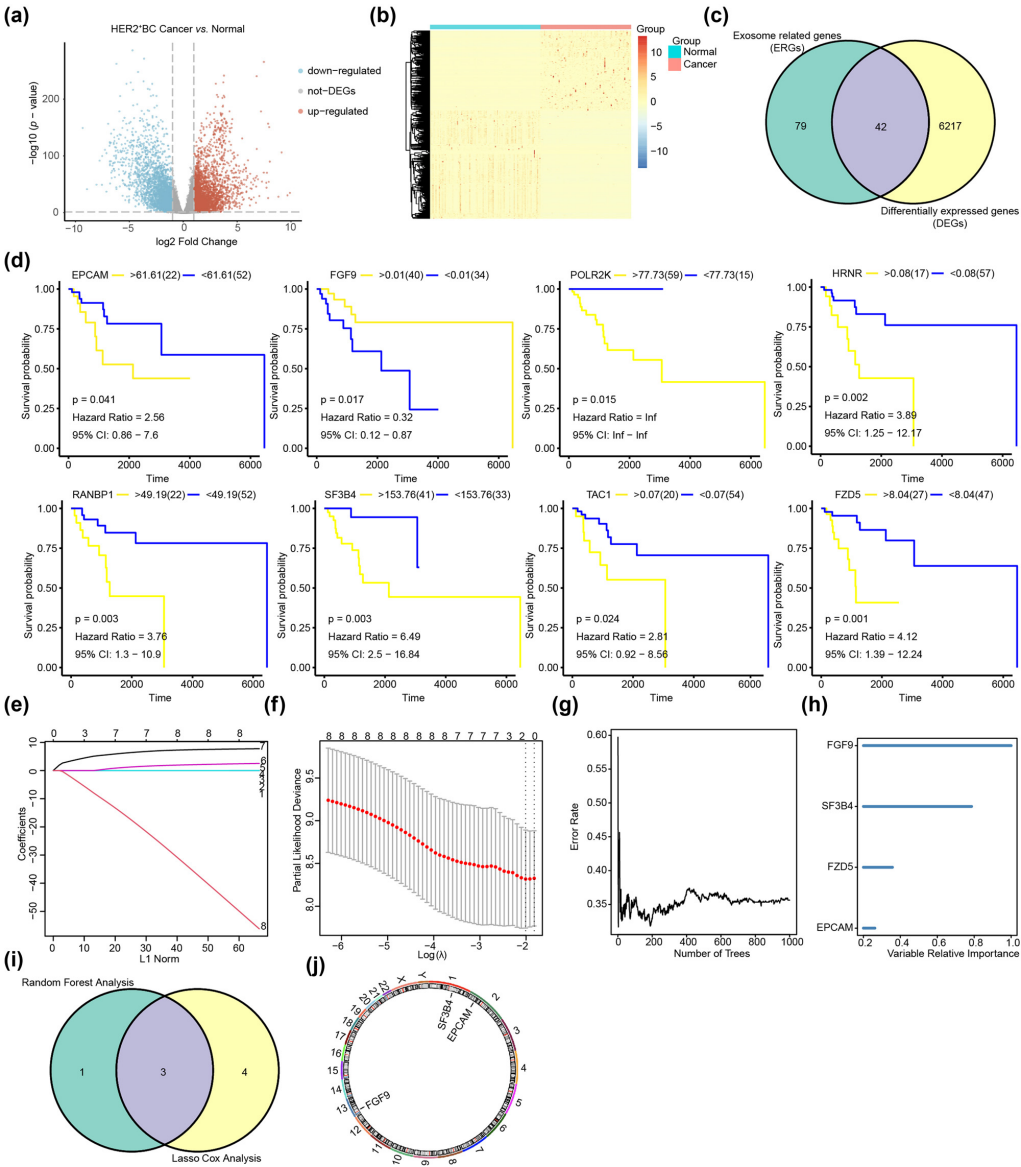
Screening of critical prognostic genes related to exosomes in patients with HER2^+^ BC. (a) DEGs in HER2^+^ BC. The abscissa of the volcanic map is log2FoldChange and the ordinate is log10 (*p*-value). The red node indicates up-regulated DEGs, the blue node represents down-regulated DEGs, and the gray node represents genes that are not significantly differentially expressed. (b) A heat map reveals DEGs in HER2^+^ BC. The blue bar indicates HER2^+^ BC samples, the pink bar indicates normal control samples, the red bar indicates high gene expression, and the blue bar indicates low gene expression. (c) The number of intersecting genes between ERGs and DEGs in HER2^+^ BC. (d) KM plots show eight ER-DEGs that exhibit significant differences in survival. (e) Diagnostic model diagram of ER-DEGs in the TCGA-BRCA dataset. (f) Variable trace plot of ER-DEGs in the LASSO-COX regression model. (g) Error rate of ER-DEGs in the random forest model. (h) Importance ranking diagram of ERGs in random forest model. (i) The Venn diagram depicting the EPGs identified in the HER2^+^ BC using random forest analysis and Lasso cox analysis. (j) Chromosomal mapping of EPGs.

The flowchart of this study is depicted in Figure 1. In cases with HER2^+^ BC from the TCGA-BRCA dataset, we found 6,259 DEGs, of which, 3,252 were upregulated and 3,006 were downregulated between breast cancer and precancerous normal samples. The blue scatter points indicate highly expressed genes, whereas the red scatter points indicate low expressed genes in HER2^+^ BC (Figure 2a). Heat maps (Figure 2b) were used to visually display the expression of each gene in HER2^+^ BC, where blue indicates low expression and red indicates high expression. Through this gradient of color, the heat map revealed significant differences in gene expression levels in HER2^+^ BC compared to precancerous normal samples. Based on the intersection of ERGs and DEGs, we found 42 ER-DEGs, which are represented in a Venn diagram (Figure 2c). We performed survival analyses on patients with HER2^+^ BC and discovered that 8 of the 42 ER-DEGs had significant survival differences – they were EPGAM (*p* = 0.041), FGF9 (*p* = 0.017), POLR2K (*p* = 0.015), HRNR (*p* = 0.002), RANBP1 (*p* = 0.003), SF3B4 (*p* = 0.003), TAC1 (*p* = 0.024), and FZD5 (*p* = 0.001) (Figure 2d). To determine the association between ER-DEGs and overall survival of patients with HER2^+^ BC, we performed the univariate Cox regression in the subgroup of HER2^+^ BC from TCGA-BRCA and discovered that 9 of the 42 ER-DEGs were associated with clinical prognosis (*p* < 0.1). We conducted additional research on the nine genes using Lasso Cox regression and the random forest algorithm. The optimal Lambda value was employed in the LASSO model (Figure 2e). To display the LASSO Cox regression outcomes, the LASSO variable trace plot was used (Figure 2f). The nine ER-DEGs were ranked using the random forest variable importance values, indicating that the error rate reflecting survival reduced with different genes (Figure 2g). To discover the genes most associated with the prognosis of HER2^+^ BC, we chose genes with an importance greater than 0.2 as HER2^+^ BC prognosis-related genes (Figure 2h). Then, we integrated the two screening models, took their intersection, and discovered three HER2^+^ BC-related prognostic genes (FGF9, SF3B4, and EPCAM) (Figure 2i) that we dubbed EPGs. We drew a chromosomal map to reveal their location on a chromosome. FGF9 is found on chromosome 13, SF3B4 is found on chromosome 1, and EPCAM is found on chromosome 2 (Figure 2j).

### 3.2 Constructing a prognostic model based on EPGs in HER2^+^ BC

**Figure 3:**
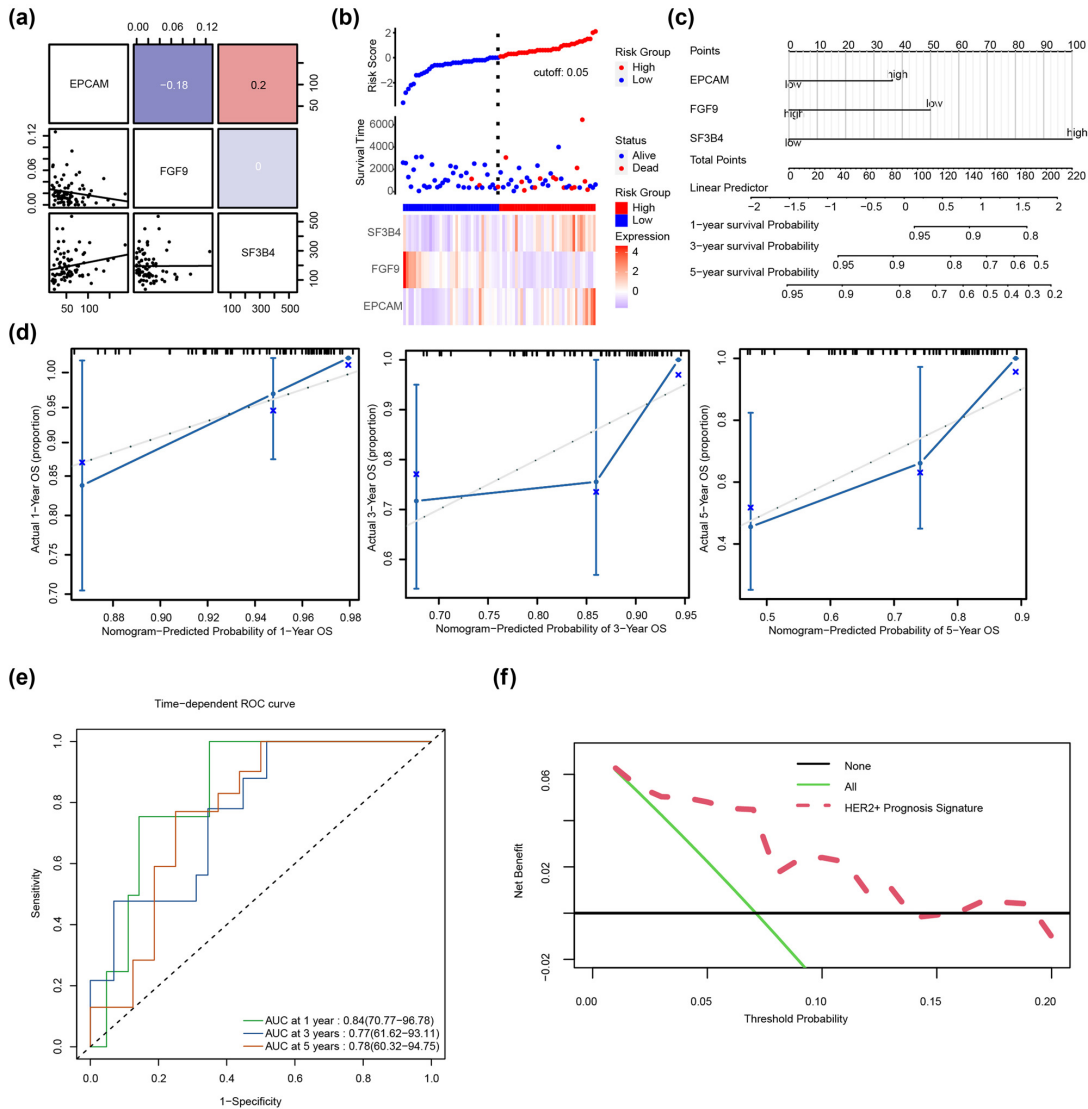
Constructing the predictive model using EPGs. (a) The link between EPGs in HER2^+^ BC. Red indicates a positive correlation, whereas blue indicates a negative correlation. (b) The relationship between EPGs and survival of patients with HER2^+^ BC. (c) The nomogram was created using Cox regression analysis of EPGs from the TCGA-BRCA dataset, specifically for HER2^+^ BC. The middle section reflects the expression levels of FGF9, SF3B4, and EPCAM, while the total score represents the total score of the patient based on the three gene scores. The total score correlates with the chance of occurrence listed below it. (d) The 1-, 3-, and 5-year recall curves revealed that the black diagonal dashed line representing the true sample situation and the blue solid line representing the predicted patient’s disease status by the prediction model overlapped in most cases. (e) The time-dependent ROC curve demonstrated the model’s ability to predict 1-, 3-, and 5-year survival. (f) The plot DCA of ERGs in the Cox regression model. The vertical axis indicates net income, and the horizontal axis represents probability threshold or threshold probability.

The correlation analysis results revealed that the correlation between EPCAM and FGF9 was −0.18, while the correlation between EPCAM and SF3B4 was 0.2. (Figure 3a). The survival of patients with HER2^+^ BC was associated with the EPGs (Figure 3b). To assess the prognostic efficacy of EPGs in patients with HER2^+^ BC, we developed a predictive prognostic model using multivariate Cox regression. We evaluated the prognostic value of the Cox regression model and illustrated the nomogram (Figure 3c), plotted the curve through calibration analysis, and evaluated the prediction effect of the model on the actual results based on the model’s fitting, to predict the 1-, 3-, and 5-year actual survival probabilities of patients with HER2^+^ BC (Figure 3d). We demonstrated the differentiation of the model in predicting the 1-, 3-, and 5-year survival probability of patients with HER2^+^ BC using time-dependent ROC curves. The results indicated that the model was optimal for predicting the 1-, 3-, and 5-year survival probabilities, with AUC values of 0.84 (70.77–96.78), 0.77 (61.62–93.11), and 0.78 (60.32–94.75). (Figure 3e). The role of the Cox regression model in clinical utility was examined using DCA (Figure 3f). When the line of the model stabilizes above All Positive and All Negative within a given range, the broader the range, the greater the net income and the better the performance of model.

### 3.3 Three subtypes recognized by EPGs in HER2^+^ BC

**Figure 4:**
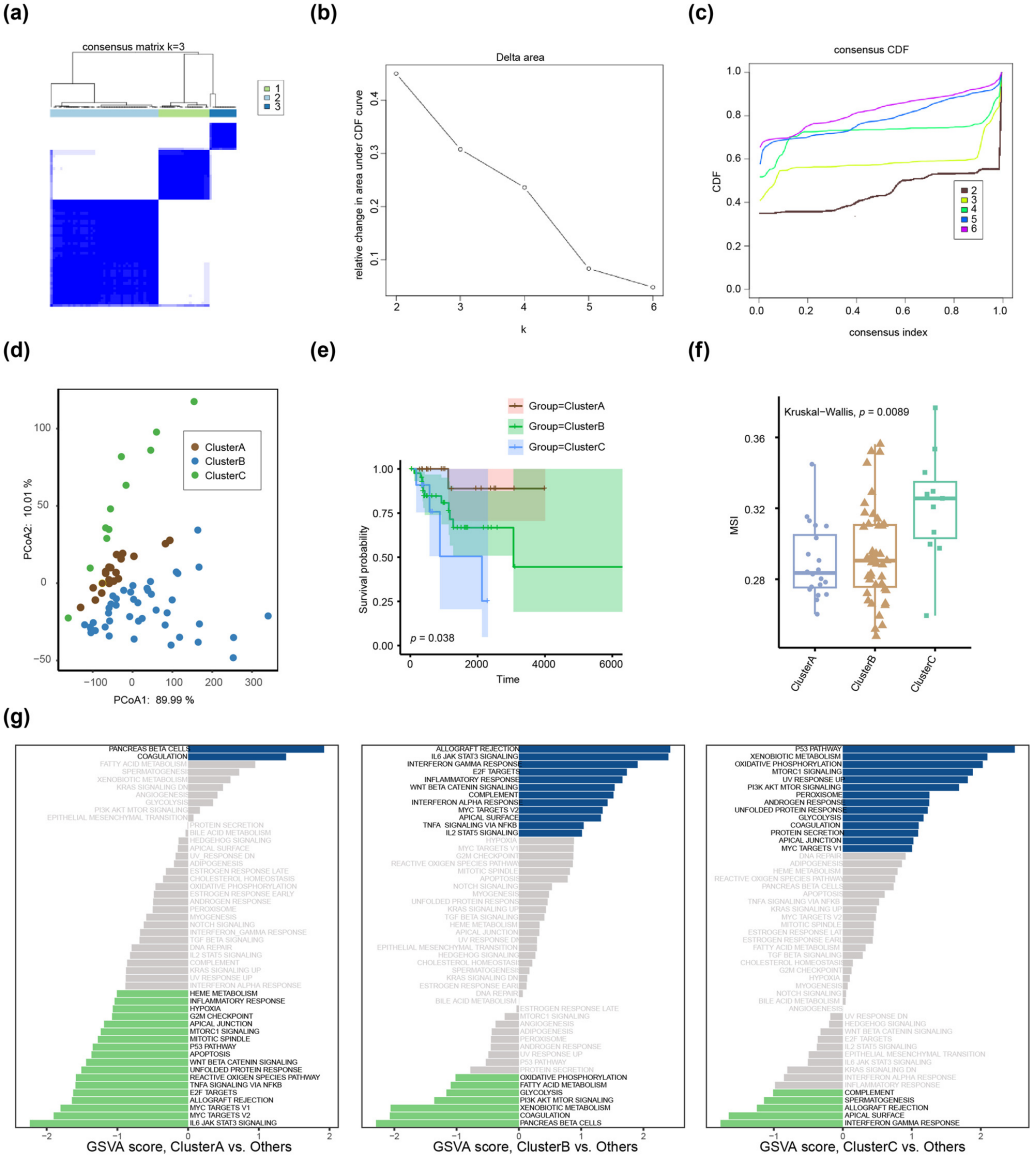
Unsupervised cluster analysis for TCGA-BRCA. (a) Consensus clustering based on EPGs (FGF9, SF3B4, and EPCAM TCGA) in the HER2^+^ BC dataset. (b) The scree plot according to cluster analysis. (c) Cumulative distribution function showed the consensus index under different *K* values. (d) PCoA analysis among the three clusters. (e) Survival curves showed the survival probability of patients in the three clusters. (f) The difference in MSI of patients in the three clusters. (g) Kyoto Encyclopedia of Genes and Genomes bifunctional enrichment analysis for Cluster A, Cluster B, and Cluster C.

We discovered three subgroups based on FGF9, SF3B4, and EPCAM using a consensus clustering approach (Figure 4a). The screen plot revealed that the optimal classification parameters could be identified using inflection points (Figure 4b). The cumulative distribution function indicated that three categories were more logical, as the rise slope of the consistency index was the lowest when consensus clustering was divided into three groups (Figure 4c). PCoA validated the features of three subtypes, and the results indicated the optimal classification stability of the three disease subtypes (Figure 4d). KM analysis revealed that the prognosis of patients with Cluster A subtype was better than that of patients with Cluster B or Cluster C with the *p*-value of 0.038 (Figure 4e). The Wilcoxon rank-sum test indicated that the degree of MSI in Cluster C was substantially greater than its degree in Cluster A (*p* < 0.05). (Figure 4f). ssGSEA analyses revealed substantially enriched biological signaling pathways in three subtypes of exonemes. The results showed that the PANCREAS BETA CELLS and COAGULATION pathways were highly enriched in Cluster A (Figure 4g). ALLOGRAFT REJECTION, IL6 JAK STAT3 SIGNALING, INTERFERON GAMMA RESPONSE, E2F TARGETS, and INFLAMMATORY RESPONSE were enriched in Cluster B (Figure 4h). P53 PATHWAY, XENOBIOTIC METABOLISM, OXIDATIVE PHOSPHORYLATION, MTORC1 SIGNALING, and UV RESPONSE UP were enriched in Cluster C (Figure 4i).

### 3.4 Mutational signatures and gene druggability of EPGs in HER2^+^ BC subgroup

**Figure 5:**
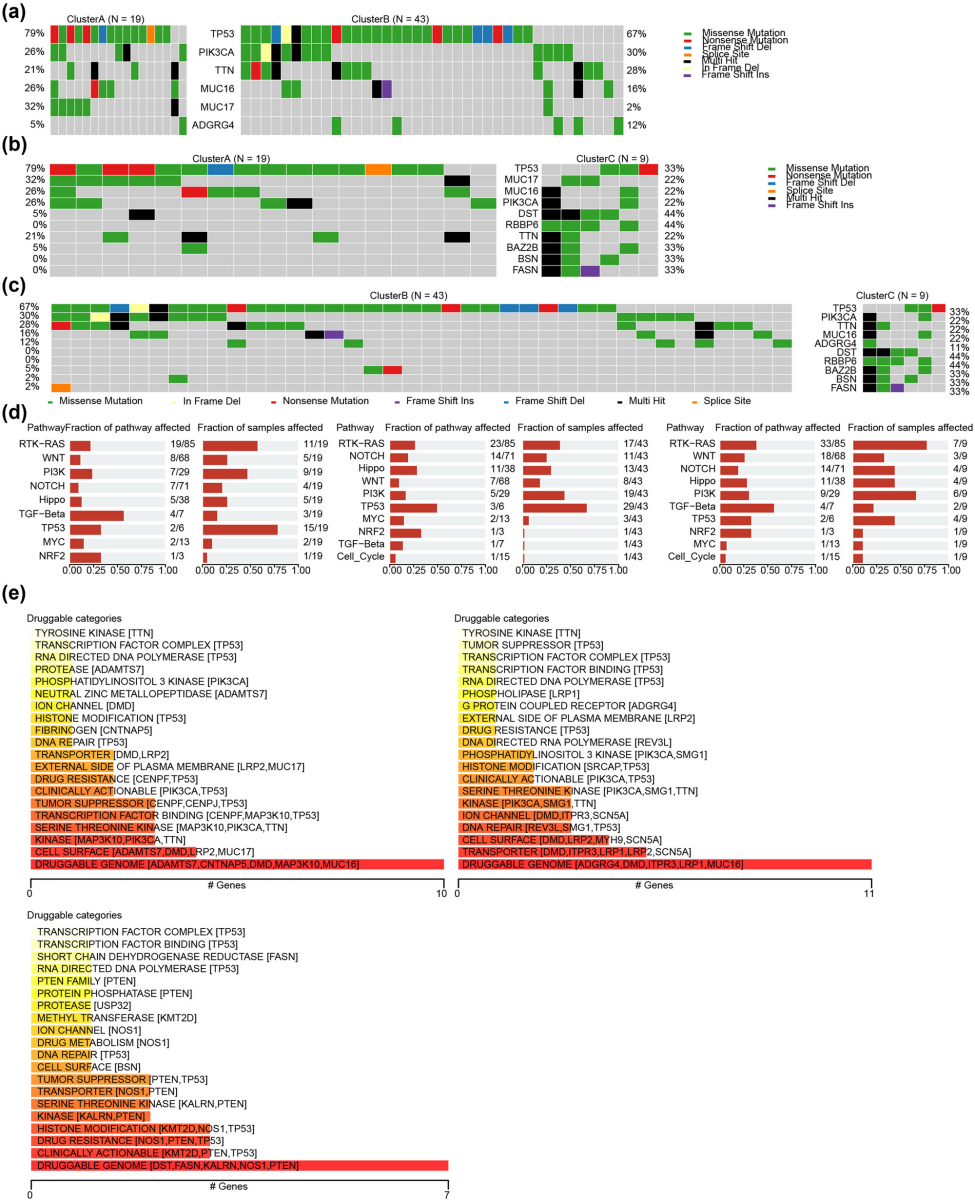
Mutation and gene druggability analysis for EPGs in the HER2^+^ BC subgroup. Comparing the landscape of mutations in ERGs among HER2^+^ BC subgroup in (a) Cluster A vs Cluster B, (b) Cluster A vs Cluster C), (c) Cluster B vs Cluster C. (d) Biological functional analysis affected by mutations in exosome-related HER2^+^ BC subgroup (left: Cluster A, middle: Cluster B; right: Cluster C). (e) The categories of potential role of druggability.

The mutation features of the exosome-related HER2^+^ BC subgroups were studied using the HER2^+^ BC in the TCGA-BRCA dataset and the R package maftools. The results showed that TP53, PIK3CA, TTN, and MUC16 had a high mutation frequency in HER2^+^ BC, of which TP53 was 79% (Figure 5a) in Cluster A, 67% (Figure 5b) in Cluster B, and 33% (Figure 5c) in Cluster C. The biological functional alterations caused by mutations in Cluster A and Cluster B were mainly concentrated in the TP53 signaling pathway, while they were mainly concentrated in the RTK-RAS signaling pathway in Cluster C subtype (Figure 5d). We explored the gene druggability and the interactions between drugs and genes based on the Drug Gene Interaction database and found that the gene predicting the possible action of drugs on patients in the Cluster A was TRANSCRIPTION FACTOR COMPLEX (TP53 et al.) (Figure 5e). The gene that predicted the potential effect of drugs on Cluster B was PHOSPHOLIPASSE (LRP1 et al.) (Figure 5f). The gene that predicted the potential effect of the drug on patients in Cluster C was SHORT CHAIN DEDROGENASE REDUCTASE (FASN et al.) (Figure 5g).

### 3.5 Immune microenvironment characteristics of EPGs in HER2^+^ BC subgroup

**Figure 6:**
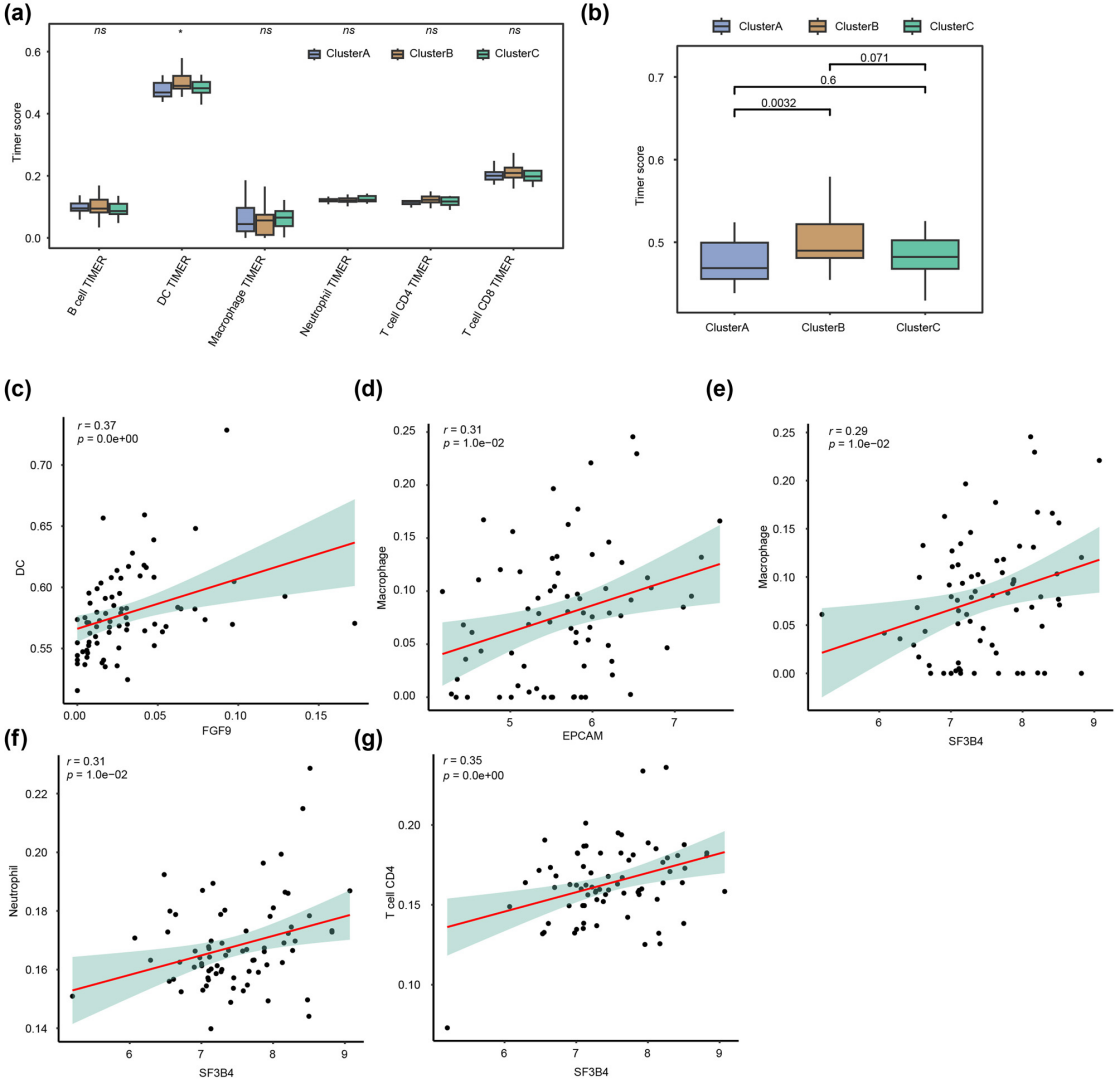
Immune infiltration analysis for EPGs in the HER2^+^ BC subgroup. (a). The box diagram of immune cell infiltration abundance in the exosome-related HER2^+^ BC subgroup. (b) Pair-wise comparisons of the box plot among the three subgroups. (c) Correlation analysis between FGF9 and DC infiltration abundance. (d) Correlation analysis between EPCAM and macrophage infiltration abundance. (e) Correlation analysis between SF3B4 and macrophage infiltration abundance. (f) Correlation analysis between SF3B4 and neutrophil infiltration abundance. (g) Correlation analysis between SF3B4 and CD4^+^ T cells infiltration abundance.

Based on the TCGA-BRCA dataset for HER2^+^ BC, we assessed the status of six types of immune cell infiltration and the immune cell infiltration among three subtypes using TIMER analysis and its algorithm. The results exhibited statistically significant differences in DC infiltration among three subtypes (*p* = 0.0001, Figure 6a). We analyzed the three subtypes in pairs and found that Cluster A differed significantly from Cluster B (*p* = 0.0032, Figure 6b). We performed correlation analyses between ERGs and the infiltration of various immune cells in HER2^+^ BC and discovered that DCs were positively correlated with the presence of FGF9-expressed instances (*r* = 0.37, *p* < 0.001, Figure 6c). Macrophages were positively correlated with the content of EPCAM expressed cases (*r* = 0.31, *p* = 0.01, Figure 6d). Macrophages (*r* = 0.29, *p* = 0.01, Figure 6e), neutrophils (*r* = 0.31, *p* = 0.01, Figure 6f), and CD4^+^ T cells (*r* = 0.35, *p* < 0.001, Figure 6g) were positively correlated with the content of SF3B4 expressed cases.

### 3.6 Validating the EPGs in 10X genomics single-cell data of BC

To validate the role of the three EPGs (EPCAM, FGF9, and SF3B4) in HER2^+^ BC in various cell types, we conducted validation analysis using 10× single cell dataset-GSE176078. Initially, we imported the count matrix of five samples from the single cell dataset using the Seurat (version 4.0.2) R package to generate the Seurat object for analysis. Based on the preliminary quality of the Cell Ranger, we did a second round of data quality control by preserving the following high-quality cells: gene numbers greater than 250, UMI numbers greater than 500, log_10_ genes per UMI greater than 0.8, mitochondrial UMI ratio less than 20%, and red blood cell gene ratio less than 5%. Then, we removed the doublet using the R package DoubletFinder. Following the previous steps, we obtained a total of 19,311 cells (Figure S1). We then standardized the sequencing depth of GSE176078 using the “Normalize Data” function, which defaults to “Log Normalize.” We identified 2,000 variable features of the dataset using the “vst” approach using the “Find Variable Features” function. Then, we used “Scale Data” to scale the data to exclude the effect of sequencing depth and used PCA with variable genes as input to identify significant principal components based on the Elbow Plot function. The top 20 principal components were chosen as statistically significant UMAP inputs.

### 3.7 Cluster analysis and cell type annotation in the single-cell dataset and scoring of EPGs in HER2^+^ BC subtypes

**Figure 7:**
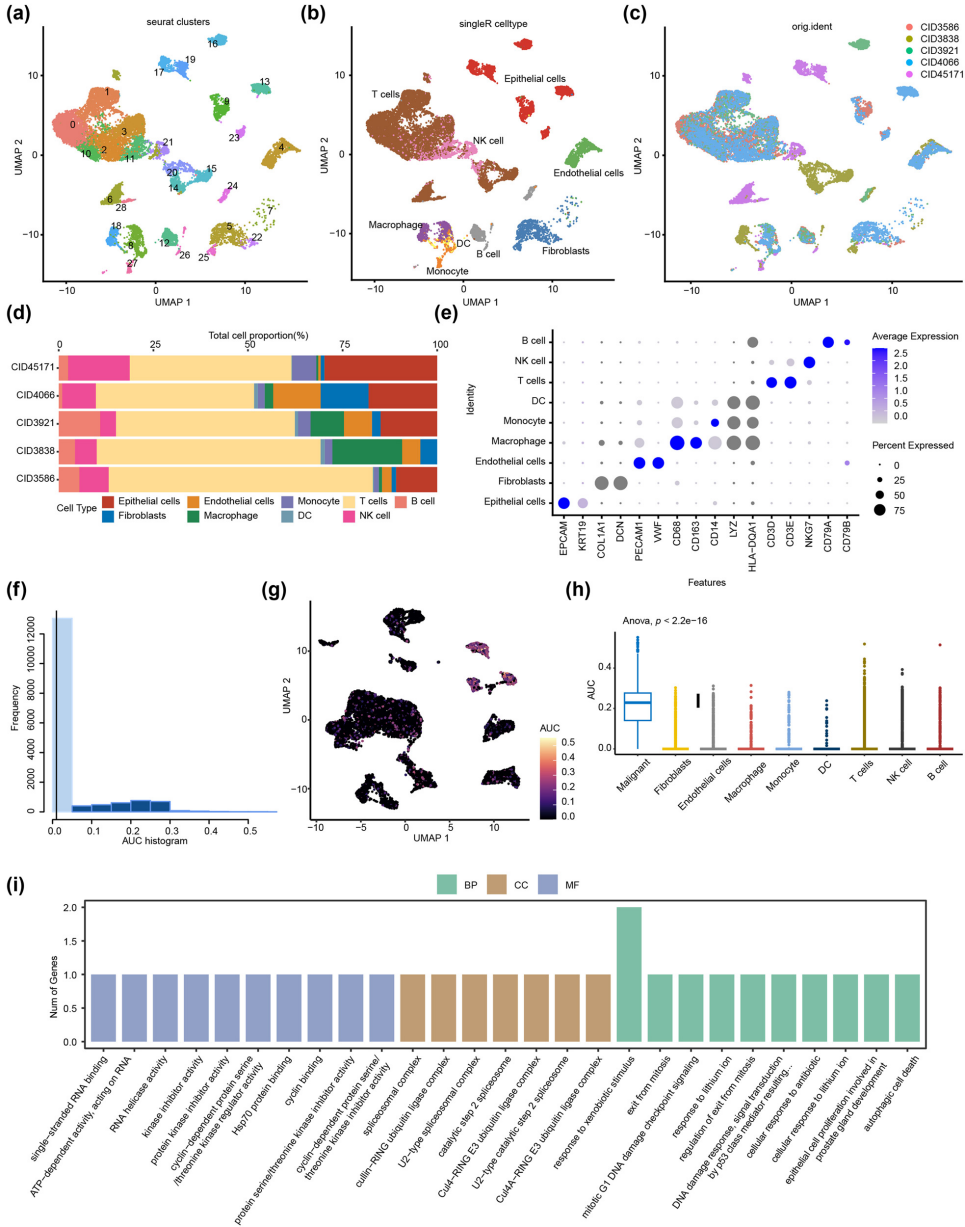
Single-cell RNA sequencing revealed the complexity of the microenvironment in HER2^+^ BC. (a) Cells were clustered into 29 clusters through UMAP based on the single cell dataset (GSE176078). (b) Cells were annotated into nine cell types through singleR and sample sources, including T cells, B cells, endothelial cells, epithelial cells, fibroblasts, monocytes, NK cells, DCs, and macrophages. (c) The UMAP diagram showed the distribution of nine cell types. (d) The bar chart displayed the proportion of each cell type in the nine cell types. (e) The heat map suggested the expression of genes specifically expressed for each cell type in the cell, with circles ranging from small to large representing the average expression value of characteristic genes. (f) The scores of the EPGs. (g) UMAP analysis in immune cells. (h) AUC box plot of EPG scores in different cell subtypes. (i) Pathway enrichment analysis of EPGs in HER2^+^ BC.

Following quality control of the HER2^+^ BC single cell dataset of GSE176078, we effectively categorized 19,311 cells into 29 independent cell clusters (Figure 7a). We annotated cells into nine distinct cell types through singleR and sample sources. Clusters 0, 1, 2, 3, 6, 10, 14, 15, and 28 were annotated as T cells (10,576, 54.77%); Clusters 12, 24, and 26 were annotated as B cells (919, 4.76%); Cluster 4 was annotated as endothelial cells (1128, 5.84%); Clusters 9, 13, 16, 17, 19, and 23 were annotated as epithelial cells (2821, 14.61%); Clusters 5, 7, 22, and 25 were annotated as fibroblasts (883, 4.57%); Cluster 27 was annotated as monocytes (477, 2.47%); Clusters 11, 20, and 21 were annotated as NK cells (1613, 8.35%); Cluster 8 was annotated as DCs (124, 0.64%); Clusters 8 and 18 were annotated as macrophages (882, 4.57%) (Figure 7b). Each sample appeared as a homogenous distribution (Figure 7c). The proportions of cell types were shown in each sample, with T cells accounting for a high proportion in each sample (Figure 7d). We screened the main marker genes of each cell type using the Cellmarker dataset and displayed them in dot plot format. EPCAM and KRT19 represent epithelial cells. PECAM1 and VWF represent endothelial cells. CD68 and CD163 represent macrophages. CD14 represents monocytes. CD3D and CD3E represent T cells. NKG7 represents NK cells. CD79A and CD79B represent B cells (Figure 7e). The DEGs between cell subtypes are listed in Table 2. Epithelial cells were classified as either malignant or non-malignant using CopyKat analysis, as depicted in Figure S2. To gain an understanding of the expression characteristics of ERGs in HER2^+^ BC, three identified EPGs were scored using the R program AUCell to compute the activity of exosome function in each cell based on the proportion of the highly expressed gene set in each cell. Cells expressing more genes from the gene set exhibit higher AUC values than those cells expressing fewer genes. We found two peaks in the AUC values of all cells. When the AUC threshold was set to 0.2, 3,434 cells showed relatively high AUC and 12,774 cells showed relatively low AUC (Figure 7f), through UMAP clustering analysis (Figure 7g) or box plot AUC comparison (Figure 7h; Figure S2, these cells are mainly distributed in malignant epithelial cells). Using gene ontology and pathway enrichment analysis to determine the functional characteristics of cell subsets in these malignant epithelial cells, we discovered that the expression properties of GRGs in HER2^+^ BC were primarily associated with RESPONSE TO XENOBIOTIC STIMULUS. The screening criteria were *p* < 0.05, and the top ten pathways were sorted by ES score (Figure 7i, Table 3)

**Table 2:** Characteristic gene information of cell type

*p*_val	avg_log2FC	pct.1	pct.2	*p*_val_adj	Cluster	Gene
0	3.5008097	0.975	0.085	0	Endothelial cells	IGFBP7
0	2.7874423	0.9	0.007	0	Endothelial cells	RAMP2
0	2.6477013	0.83	0.003	0	Endothelial cells	PLVAP
0	2.6393137	0.87	0.051	0	Endothelial cells	SPARCL1
0	2.5954879	0.99	0.302	0	Endothelial cells	IFITM3
0	2.5215686	0.51	0.002	0	Endothelial cells	ACKR1
0	2.5190479	0.943	0.1	0	Endothelial cells	IFI27
0	2.1214615	0.875	0.035	0	Endothelial cells	RNASE1
0	2.0813175	0.836	0.027	0	Endothelial cells	HSPG2
0	2.0796495	0.897	0.114	0	Endothelial cells	IGFBP4
0	3.0812194	0.625	0.047	0	NK_cell	GNLY
0	2.2828379	0.759	0.141	0	NK_cell	NKG7
0	1.8853469	0.525	0.066	0	NK_cell	XCL1
0	1.8804565	0.485	0.05	0	NK_cell	XCL2
0	1.5454339	0.42	0.052	0	NK_cell	GZMB
0	1.2911218	0.809	0.33	0	NK_cell	CCL5
0	1.2029094	0.672	0.144	0	NK_cell	CTSW
0	1.1969762	0.592	0.057	0	NK_cell	KLRD1
0	1.0595189	0.678	0.205	0	NK_cell	CST7
0	1.777129	0.607	0.189	0	NK_cell	CCL4
0	5.3961354	0.925	0.021	0	Fibroblasts	COL1A1
0	5.1014286	0.946	0.02	0	Fibroblasts	COL1A2
0	4.8643579	0.931	0.014	0	Fibroblasts	COL3A1
0	4.6323219	0.909	0.007	0	Fibroblasts	SFRP2
0	4.2776688	0.93	0.008	0	Fibroblasts	LUM
0	4.0859518	0.94	0.07	0	Fibroblasts	SPARC
0	4.0236418	0.946	0.007	0	Fibroblasts	DCN
0	3.9159311	0.809	0.013	0	Fibroblasts	POSTN
0	3.8171428	0.66	0.006	0	Fibroblasts	MMP11
0	3.5036856	0.853	0.022	0	Fibroblasts	CTHRC1
0	5.5917694	0.971	0.08	0	Macrophage	APOE
0	4.4926105	0.933	0.029	0	Macrophage	APOC1
0	4.1681647	0.542	0.024	0	Macrophage	SPP1
0	4.0036169	0.999	0.261	0	Macrophage	HLA-DRA
0	4.0025266	0.977	0.019	0	Macrophage	C1QB
0	3.9784419	1	0.958	0	Macrophage	FTL
0	3.8855317	0.985	0.02	0	Macrophage	C1QA
0	3.782816	0.98	0.017	0	Macrophage	C1QC
0	3.6845234	0.981	0.208	0	Macrophage	CTSB
0	3.680925	0.957	0.039	0	Macrophage	LYZ
0	4.5888552	0.305	0.023	0	B_cell	IGHG1
0	3.6141401	0.168	0.003	0	B_cell	IGHG2
0	2.822074	0.34	0.007	0	B_cell	JCHAIN
0	4.5251031	0.119	0.003	0	B_cell	IGHV3-23
0	4.3981597	0.265	0.031	0	B_cell	IGHG3
0	3.1499148	0.245	0.032	0	B_cell	IGHA1
0	5.7093188	0.26	0.038	0	B_cell	IGLC2
0	5.2700108	0.357	0.075	0	B_cell	IGKC
0	6.6128799	0.134	0.014	0	B_cell	IGLV2-14
0	4.5139674	0.167	0.025	0	B_cell	IGLC3
0	1.6121143	0.706	0.101	0	T_cells	IL7R
0	1.4663435	0.857	0.36	0	T_cells	RGCC
0	1.3521761	0.987	0.695	0	T_cells	BTG1
0	1.3504311	0.808	0.269	0	T_cells	TNFAIP3
0	1.2750278	0.859	0.451	0	T_cells	TSC22D3
0	1.2696695	0.901	0.577	0	T_cells	SARAF
0	1.2689862	0.775	0.086	0	T_cells	CD3E
0	1.1577115	0.577	0.108	0	T_cells	LTB
0	1.145201	0.684	0.049	0	T_cells	CD3D
0	1.1788459	0.634	0.472	0	T_cells	ANXA1
0	3.2032857	0.868	0.061	0	Monocyte	LYZ
0	2.7284108	0.979	0.277	0	Monocyte	HLA-DRA
0	2.6981768	0.977	0.216	0	Monocyte	CST3
0	2.5125578	0.589	0.021	0	Monocyte	IL1B
0	2.426797	0.927	0.109	0	Monocyte	TYROBP
0	2.3950507	0.374	0.035	0	Monocyte	S100A8
0	2.8961996	0.479	0.062	0	Monocyte	S100A9
0	2.6940977	0.881	0.301	0	Monocyte	HLA-DPA1
0	2.6823252	0.886	0.312	0	Monocyte	HLA-DPB1
0	2.5512792	0.984	0.662	0	Monocyte	CD74
0	3.0065951	0.83	0.076	0	DC	LYZ
0	2.872482	0.955	0.195	0	DC	HLA-DQA1
0	3.1076022	0.955	0.23	0	DC	CST3
0	3.3514534	0.991	0.29	0	DC	HLA-DRA
0	3.3879318	0.982	0.312	0	DC	HLA-DPA1
0	3.3525582	0.982	0.322	0	DC	HLA-DPB1
0	3.1123536	0.991	0.36	0	DC	HLA-DRB1
0	2.6186613	0.429	0.05	0	DC	G0S2
0	3.1815724	1	0.668	0	DC	CD74
0	2.7433919	0.705	0.192	0	DC	HLA-DRB5
0	4.899681	0.439	0.077	0	Epithelial cells	MUCL1
0	4.1936505	0.314	0.028	0	Epithelial cells	SCGB2A2
0	3.2109667	0.519	0.02	0	Epithelial cells	CALML5
0	2.8713105	0.62	0.143	0	Epithelial cells	MIEN1
0	2.8496767	0.871	0.031	0	Epithelial cells	CD24
0	2.826804	0.875	0.027	0	Epithelial cells	KRT7
0	2.8203469	0.607	0.01	0	Epithelial cells	KRT19
0	2.723252	0.548	0.111	0	Epithelial cells	MGP
0	2.6634161	0.796	0.395	0	Epithelial cells	DBI
0	5.4093452	0.272	0.088	0	Epithelial cells	SCGB1B2P

**Table 3:** Enrichment analysis results of DEGs in cell clusters of high malignant epithelial cell

Ontology	ID	Description	Gene ratio	Bg ratio	*p* value	*p*.adjust	*q* value	gene ID	Count
CC	GO:0005743	Mitochondrial inner membrane	8/67	491/19594	0.0002619	0.0335883	0.0263503	NME4/TIMM13/MRPS34/NDUFB9/COX6C/MRPL14/SLC25A39/UQCRQ	8
CC	GO:0098798	Mitochondrial protein-containing complex	6/67	281/19594	0.0003954	0.0335883	0.0263503	TIMM13/MRPS34/NDUFB9/COX6C/MRPL14/UQCRQ	6
CC	GO:0071005	U2-type precatalytic spliceosome	3/67	50/19594	0.0006677	0.0335883	0.0263503	SF3B4/LSM4/SNRPE	3
CC	GO:1990204	Oxidoreductase complex	4/67	120/19594	0.0007615	0.0335883	0.0263503	NDUFB9/ETFA/UQCRQ/P4HB	4
CC	GO:0071011	Precatalytic spliceosome	3/67	53/19594	0.0007922	0.0335883	0.0263503	SF3B4/LSM4/SNRPE	3
CC	GO:0016328	Lateral plasma membrane	3/67	64/19594	0.0013717	0.0484654	0.0380215	EPCAM/TACSTD2/CLDN4	3
MF	GO:0016209	Antioxidant activity	4/64	85/18410	0.0002177	0.042997	0.0370511	MGST1/TXN/PRDX2/PRDX4	4
MF	GO:0016860	Intramolecular oxidoreductase activity	3/64	50/18410	0.0006989	0.042997	0.0370511	MIF/P4HB/DDT	3
MF	GO:0004601	Peroxidase activity	3/64	52/18410	0.0007842	0.042997	0.0370511	MGST1/PRDX2/PRDX4	3
MF	GO:0016684	Oxidoreductase activity, acting on peroxide as acceptor	3/64	56/18410	0.0009739	0.042997	0.0370511	MGST1/PRDX2/PRDX4	3
MF	GO:0016863	Intramolecular oxidoreductase activity, transposing C═C bonds	2/64	14/18410	0.0010538	0.042997	0.0370511	MIF/DDT	2

### 3.8 Cell–cell communication analysis

**Figure 8:**
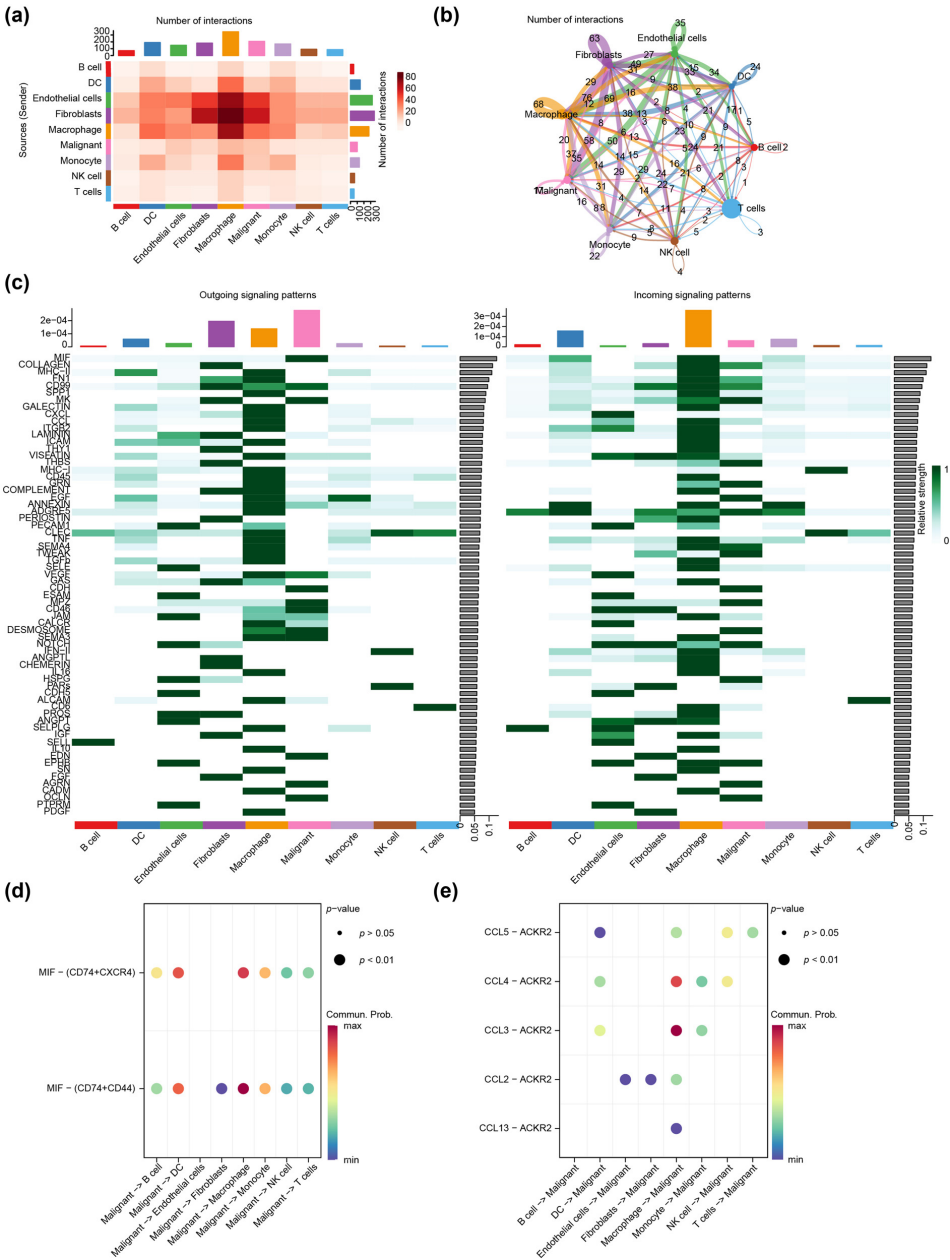
Cell–cell communication analysis. (a) The heatmap revealed the intensity of cell–cell communication including T cells, B cells, endothelial cells, epithelial cells, fibroblasts, monocytes, NK cells, DCs, and macrophages based on the GSE176078 dataset. (b) Circular graph exhibits cell communication numbers. (c) The heat map indicating contribution values of pathway involved in output and input, with the green color representing the intensity of correlation between the cells with each other. (d) and (e) Bubble diagram showing the cell–cell communication (d) received and (e) emitted by malignant epithelial cells. The bubble size represents the *p*-value, and the color represents the cell–cell communication probability.

We inferred and quantified communication between nine cell types using CellChat, and visualized cell communication intensity and quantity using heat maps (Figure 8a) and circular plots (Figure 8b), which indicated a relatively high interaction intensity between malignant epithelial cells and fibroblasts or endothelial cells or monocytes, as well as a relatively high number of interactions between malignant epithelial cells and fibroblasts or endothelial cells. We also systematically investigated the cell–cell communication network to identify the two pathways that contribute the most to the outgoing or incoming signals of malignant epithelial cells (Figure 8c). Migration inhibitory factors (MIF) pathway contributes most to input, whereas C-C motif chemokine ligand (CCL) pathway contributes most to output. We demonstrated the cellular communication received by malignant epithelial cells (Figure 8d) and emitted by malignant epithelial cells (Figure 8e) via the MIF pathway and CCL pathway through bubble diagrams. In the cellular communication received by malignant epithelial cells, macrophages were the major immune cell in both, in the MIF pathway engaged in incoming cell–cell communication and in the CCL pathway engaged in outgoing cell–cell communication.

## 4 Discussion

Although ERGs have been identified as diagnostic and prognostic indicators for breast cancer [37], their potential clinical importance in HER2^+^ BC is uncertain. Herein, eight genes were extracted via deferentially expressed genes (normal vs tumor) of TCGA BRCA dataset and ERGs were merged and KM analysis was utilized to obtain eight prognostic genes. As higher infiltration of immune cells and expression of immune activation markers are seen in HER2^+^ BC primary lesions compared to metastatic tissues [38], we attempted to determine the relationship between exosomes and prognosis or microenvironment in patients with HER2^+^ BC. By combining diverse expression analysis, prognostic relevance, and ERGs, we determined the candidates for EPGs. After LASSO Cox analysis and random forest cox analysis, we finally identified FGF9, SF3B4, and EPCAM as the EPGs for HER2^+^ BC.

In the context of examining the expression profile of EPGs in HER2^+^ BC, “EPGs” specifically refers to the three genes we identified as critically important: FGF9, SF3B4, and EPCAM. These genes showed a clear association with the immune response to foreign stimuli, underscoring their potential role in regulating the tumor microenvironment in HER2^+^ BC through an exosome-mediated mechanism. FGF9, the protein produced by the fibroblast growth factor 9, acts as a secreted factor that binds fibroblast growth factor receptor and inhibits growth stimulation in the microenvironment [39]. In contrast to our findings, Xu et al. discovered that patients with ovarian cancer had decreased FGF9 expression and displayed favorable prognostic values. Abnormally produced FGF9 was associated with immunological markers, such as immunoinhibitory and chemokine receptors in ovarian cancer [40]. Our results indicated that patients with HER2^+^ BC and low FGF9 expression experienced poor survival. EPCAM encodes an antigen related to carcinoma called epithelial cell adhesion molecule, which is expressed in malignant epithelial tumors. EPCAM is used as a target for immunotherapy in the treatment of various carcinomas [41]. Although EPCAM serves as the marker protein for enriching tumor-derived exosomes, Rupp et al. discovered a reduction of EPCAM expression in serum exosomes produced from patients with breast cancer [42]. Leblanc et al. demonstrated that immunocapture of breast cell exosomes by targeting EPCAM was blocked by syntenin [43]. We discovered a negative correlation between EPCAM and FGF9 expression in HER2^+^ BC. SF3B4 encodes one of four subunits of the splicing factor 3B and is involved in pre-mRNA splicing [44]. Its function in exosomes and clinical importance in breast cancer are well understood. Our research demonstrated that SF3B4 had prognostic value in HER2^+^ BC.

We subtyped all HER2^+^ BC cases into three clusters and discovered that Cluster C had a relatively high level of MSI and p53 signaling pathway genes. Pizzi et al. demonstrated that p53 was downregulated in breast cancer characterized by MSI [45]. Dutta et al. discovered that breast cancer cells stimulate DNA damage repair and phosphorylation of p53 in mammary epithelial cells by secreting exosomes that are taken up by these cells, thereby creating a favorable environment for breast cancer [46]. The results of our study suggests that patients with HER2^+^ BC subtyped into Cluster C have a high frequency of TP53 mutations, whereas the biological functional abnormalities produced by mutations in Cluster A and Cluster B were primarily centered in the TP53 signaling pathway. Beis et al. proposed that potential new drug targets included genes that were differentially expressed in patients with various prognostic profiles or drug administration [47]. Domenis et al. discovered that the activation of pro-inflammatory cytokines increased the transfer of mutated p53 from tumor cells to epithelial cells via exosomes, thereby inserting the effect on malignant transformation and tumor progression [48]. Our research demonstrated that p53 may be the target that Cluster A drugs act upon. Zhou et al. reported that exosomal LRP1 promoted the migration of ovarian cancer cells, whereas Ricklefs discovered that FASN was elevated in malignant glioma cells and extracellular vesicles of plasma [49,50]. We believe that LRP1 and FASN are the drug-related targets of Cluster B and Cluster C.

DCs are antigen-presenting cells with multiple functions that contribute to the initiation and modulation of innate and adaptive immune responses. Romagnoli et al. suggested that CD3^+^ T cells cultured with DCs derived exosome-treated breast cancer cells, which exhibited a significantly higher proportion of IFN-γ-secreting cells, indicating that incorporation of DC-derived exosomes by the breast cancer cells could activate T-cells for a potentially more effective cancer immunotherapy [51]. It is generally recognized that DC-based treatments, particularly DC vaccination, have a therapeutic effect on breast cancer [52]. Our research revealed that the infiltration of DCs varied over three clusters, and that the expression of FGF9 was strongly linked with DCs.

Wu performed scRNA Seq (Chromium, 10X Genomics) on 26 primary tumors from three major clinical subtypes of breast cancer, including 11 ER^+^ BC, 5 HER2^+^ BC, and 10 triple-negative breast cancer; this allowed us the opportunity to investigate the involvement of EPGs in the microenvironment for subtypes of HER2^+^ BC [53]. The functions of EPGs may be primarily associated with RESPONSE TO XENOBIOTIC STIMULUS, indicating that exosomes play a vital role in the cellular response to external stimulation.

CCL family and macrophage MIFs play a significant role in breast cancer progression and its interaction with the microenvironment. Breast cancer cells promote tumor-derived CCL20 and upregulate PD-L1 expression on neutrophils to exacerbate T cell immunosuppression [54]. Liu et al. discovered that exosomes produced from mesenchymal stem cells mediate heart healing following myocardial infarction through the upregulation of MIF [55]. Li et al. found that adipose-derived stem cells interact with macrophages and accelerate bone repair via downregulation of macrophage MIF in exosomes [56]. Our research suggests that HER2^+^ BC cells interacted mostly with macrophages via MIF and CCL signaling pathway by exosomes. However, our study contains some limitations. The detected EPGs necessitate functional investigation in the context of the immunological microenvironment of HER2^+^ BC, and the HER2^+^ BC clinical samples must be confirmed using scRNA sequencing. There are also intrinsic biases in bioinformatics analyses. Despite these limitations, our approach provides bioinformatic hints for further research.

## 5 Conclusion

Our study reveals that exosomes from HER2^+^ BC patients have significant prognostic value and successfully identified EPGs (FGF9, SF3B4, and EPCAM), which were significantly associated with patient survival. In addition, our analysis highlighted the complex interactions between tumor and microenvironment, revealing the mechanism of information transmission through exosomes, which are not only critical for tumor growth and progression, but also have a profound impact on treatment response and patient prognosis.

## Supplementary Material

supplementary material
