# Supplementary material for: Prognostic value and microenvironmental crosstalk of exosome-related signatures in human epidermal growth factor receptor 2 positive breast cancer
Source: Open Life Sci. 2024 Jul 24;19(1):20220899. doi: 10.1515/biol-2022-0899 (PMC11282918; doi:10.1515/biol-2022-0899)
Supplement: supplementary material [file biol-2022-0899-sm.pdf]

Supplementary material

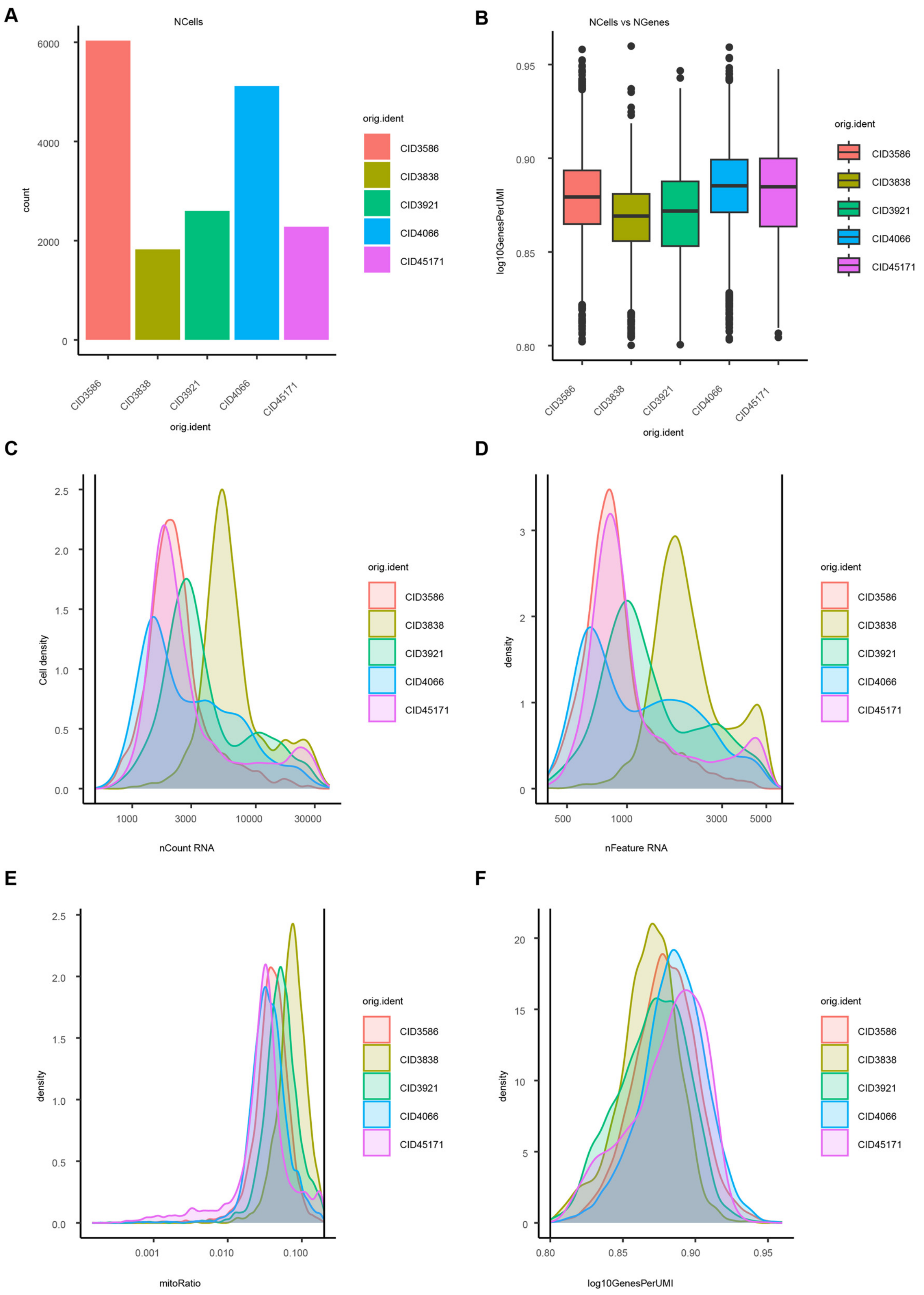

Figure S1: Seurat controls the quality of single cell data. (a) Number of gene entries, (b) the values of log10 gene entries, (c) RNA count, (d) RNA concentration (e) mitochondrialdensity, and (f) the density of the gene number taking the value of log10 were obtained by single cell sequencing technology in five HER2+ BC samples.

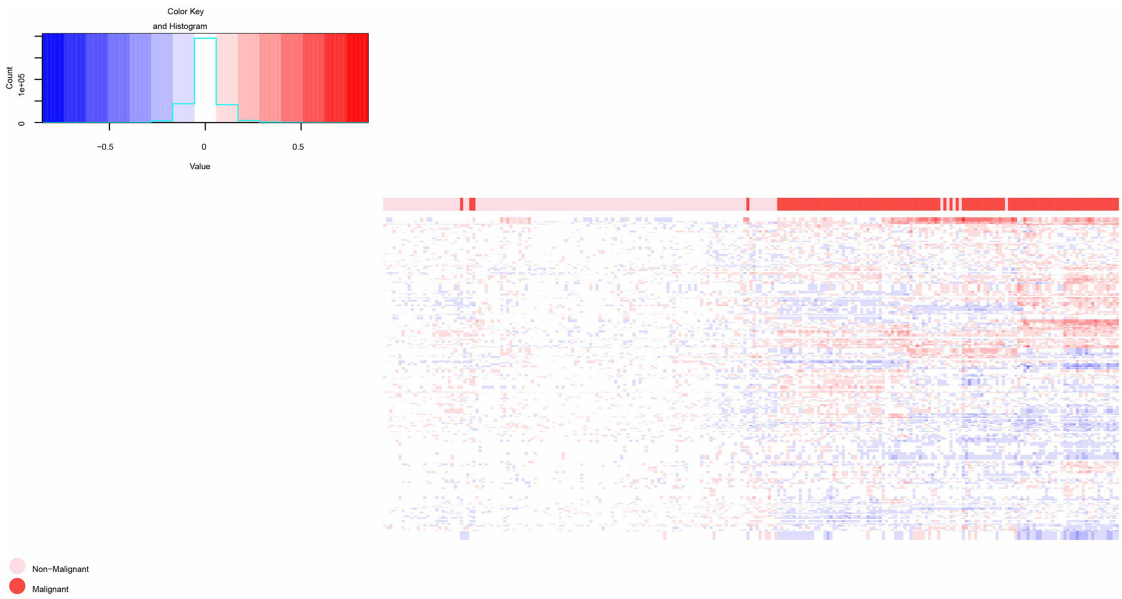

Figure S2: Determining malignant tumor cells through CNV level using CopyKat analysis. In the heat map, the red bar represents CNV amplification while the blue bar represents CNV deletion. In the heat map legends, the red bar represents malignant tumor cells, while the pink bar represents normal epithelial cells.

Table S1: Exosome related genes

Genes
CDC42, CP, CD47, EEF1A1, EPCAM, ERBB2, FGFR2, CHEK2, BARD1, TSC2, CASP7, SFN, TYR, CCKBR, CYP11A1, GATA4, ABCB11, IVL, BID, SDHA, USF1, RHO, TAC1, HLA-DQA1, RIPK1, CUL4A, ABCB5, ADCYAP1, FZD5, RANBP1, CASC3, GPBAR1, PSMC5, POLR2K, PLCB4, DOK1, SF3B4, GTF2I, NOC2L, MEF2A, CYC1, MRPL21, MRPL4, BDKRB2, PIK3R5, PRPF3, PSMD2, SNRPA1, RPS10, ADRA1B, TRIOBP, RPL26L1, FAM162A, SF3A2, HER2, CD82, CD24, Del-1, Dicer, POSTN, FGFR3, FIBP, FRS2, FTH1, FTL, GSTP1, HOXC6, HTR7, IGF1R, IL1RAP, ITGB1, KISS1R, KRT14, LDHA, MTDH, NANOG, NEUROD1, Plau, RAB13, RPL28, S1PR2, TGFBR1, TRPC5, TSG101, UCHL1, ADAM10, CYP19A1, CTSB, ELK1, PIGR, FGF9, DUSP1, MRPS30, DMRT1, CXCL13, BLZF1, ANAPC1, ALDH9A1, ENPP1, CDC23, ARF6, HDC, CSTB, POU4F1, TLR5, ABCC9, RBM10, DTX3L, ADH1A, HEXA, MRPL15, ITGB7, DCT, HRNR, CACNB2, AP2S1, DOK7, PKN2, TPP2, ABCB1, BIRC5
